# Focused Ultrasound Treatment of a Spheroid In Vitro Tumour Model

**DOI:** 10.3390/cells11091518

**Published:** 2022-04-30

**Authors:** Lisa Landgraf, Adam Kozlowski, Xinrui Zhang, Marc Fournelle, Franz-Josef Becker, Steffen Tretbar, Andreas Melzer

**Affiliations:** 1 Innovation Center Computer Assisted Surgery, Institute at the Faculty of Medicine, Leipzig University, 04103 Leipzig, Germany; lisa.landgraf@medizin.uni-leipzig.de (L.L.); adam.kozlowski@uwm.edu.pl (A.K.); xinrui.zhang@medizin.uni-leipzig.de (X.Z.); 2 Department of Anatomy, School of Medicine, Collegium Medicum, University of Warmia and Mazury, 10-082 Olsztyn, Poland; 3 Fraunhofer Institute for Biomedical Engineering (IBMT), 66280 Sulzbach, Germany; marc.fournelle@ibmt.fraunhofer.de (M.F.); franz.josef.becker@ibmt.fraunhofer.de (F.-J.B.); steffen.tretbar@ibmt.fraunhofer.de (S.T.)

**Keywords:** focused ultrasound, FUS, spheroid, in vitro experiments, prostate cancer, glioblastoma

## Abstract

**Simple Summary:**

Ultrasound waves can be applied for diagnostic and therapeutic purposes. Focused ultrasound is approved for tissue ablation, e.g., in the treatment of uterine fibroids or essential tremors. Besides the non-invasive image-guided surgical intervention at temperatures above 55 °C, FUS is investigated in other fields like blood-brain barrier opening, hyperthermia, and neuromodulation. FUS offers potential as an adjuvant therapy in cancer treatment. Therefore, analysis of FUS effects on cancer cells is necessary. We performed studies on two human cancer cell line spheroids using a newly developed high-throughput in vitro FUS applicator with 32 individual transducers. This study aimed to perform basic experiments with a new in vitro FUS device on a 3D tumour model to acquire insight into the effects of FUS at the cellular level. These experiments may contribute to a better understanding and predictions of cancer treatment efficacy.

**Abstract:**

Focused ultrasound (FUS) is a non-invasive technique producing a variety of biological effects by either thermal or mechanical mechanisms of ultrasound interaction with the targeted tissue. FUS could bring benefits, e.g., tumour sensitisation, immune stimulation, and targeted drug delivery, but investigation of FUS effects at the cellular level is still missing. New techniques are commonly tested in vitro on two-dimensional (2D) monolayer cancer cell culture models. The 3D tumour model—spheroid—is mainly utilised to mimic solid tumours from an architectural standpoint. It is a promising method to simulate the characteristics of tumours in vitro and their various responses to therapeutic alternatives. This study aimed to evaluate the effects of FUS on human prostate and glioblastoma cancer tumour spheroids in vitro. The experimental follow-up enclosed the measurements of spheroid integrity and growth kinetics, DNA damage, and cellular metabolic activity by measuring intracellular ATP content in the spheroids. Our results showed that pulsed FUS treatment induced molecular effects in 3D tumour models. With the disruption of the spheroid integrity, we observed an increase in DNA double-strand breaks, leading to damage in the cancer cells depending on the cancer cell type.

## 1. Introduction

In the management of cancerous disease, modern minimal or non-surgical strategies like radiofrequency, microwave ablation, or focused ultrasound (FUS) are used to ablate the tissue for the direct destruction of cancer cells. FUS can be applied to sensitise them by heating (41–47 °C) as an adjuvant therapy [1] to other treatment modalities, including chemo and/or radiation therapy. In this context, FUS brings value with its non-invasive, incision-free, and ionising-free treatment characteristics, controllability via real-time MR image guidance, and the capacity to activate the immune system [2].

In developing new cancer treatment strategies or new drugs, the process routinely starts with cancer cells in standardised culture conditions in vitro using a two-dimensional (2D), homogeneous model [3]. The method is advantageous in terms of its wide availability, ease of use, and low costs. In such a scenario, a singular cell suspension is distributed on a flat plate surface, where further investigative manipulations are performed to analyse the potential therapeutic agent effect. This given design inadequately reflects the in-vivo cellular arrangement and structure, which might also risk being expressed when translating into the clinical environment, producing a lack of success in achieving desired and adequate results. A more realistic 3D cell model is an initial step required to create more lifelike and complex in-vitro models. This not entirely eliminates but significantly improves the in vitro model scope and limitations. The artificial format restrains the reproduction of tissue and systems-level cellular interactions, preventing and maintaining accurate physiological processes as found occurring in a tumour’s natural in-vivo habitat.

In our study, spherical (3D) homogenous cancer aggregates were an entry point in establishing in-vivo imitating tumour systems. Three-dimensional cell cultures reveal features that cell monolayer models lack, such as an advanced network of the cell-cell complexity with the development of pH, oxygen, and metabolic gradients—stratifying mature spheroids to develop a secondary necrotic core and proliferation zone [4], resembling the avascular stage of solid tumours and actively circulating cancer cells (micro-metastases). Investigation of such 3D model behaviour in response to various treatment modalities is superior to the previously mentioned 2D setting and serves as a better scientific approach. Peripheral cells of such a model resemble the situation of actively circulating tumour cells being adjacent to capillaries in the in vivo state. In contrast, the innermost ones eventually die due to hypoxic conditions via apoptosis or necrosis, forming a secondary necrotic core [5]. Oxygenic stress is an important aspect of many physiological processes embraced in numerous human diseases, including cancer. Notably, hypoxic areas in tumours often lead to lower efficiency of radio-therapies [6]. The investigation of hypoxia is challenging to establish due to the low availability of sensitive fluorescent dyes [7], explicitly measuring the activity of hypoxic cells.

Many novel 3D organotypic models have been introduced in recent years. These sophisticated cancer cell models resemble tissue structure, function, and even disease progression. Furthermore, 3D multicellular tumour spheroid co-cultures, combining different, e.g., immune cells with cancer cells, are being created [4], resembling one of the more realistic approaches in tumour modelling. 3D tumor models have been recently noted for efficient evaluating the cancer ability or therapeutic efficiency [8] such as proliferation [9], alternation [10], invasion [11], morphology [12], or drug resistance [13]. These systems enabled the promotion of the development of new drug candidates or novel therapeutic effect [8]. However, there are few papers on the application of FUS on tumor spheroids mainly in the context of nanoparticle formulations [14,15].

Furthermore, DNA double-strand breaks (DSBs) are conducive to both genomic instability and cancer treatment. Monitoring DNA damage in a cell by detecting immunolabelled *γ*H2A.X is useful to track cancer progression and treatment effects [16]. Flow cytometry, to rapidly quantify *γ*H2A.X, allows for attaining the profile of tumour behaviour under therapeutic stress. Another classical investigative endpoint between treated and untreated spheroids relies on observing the cell survival and spheroid growth delay, including integrity and volume loss [17].

**Figure 1:**
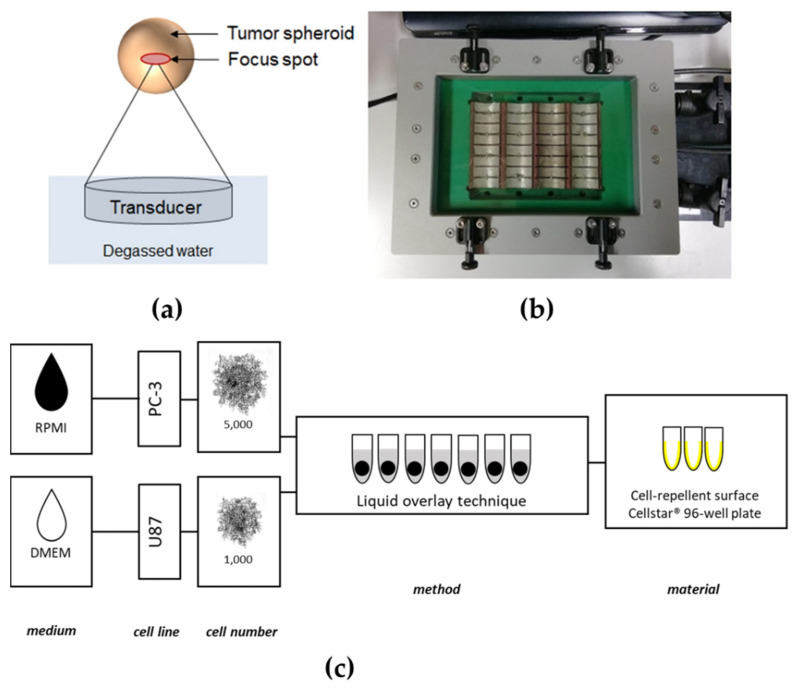
FUS treatment system and generation process of tumour spheroids. (**a**) Scheme of the in vitro FUS transducer and spheroid treatment. (**b**) The FUS in vitro system was specially designed for 96-well cell culture plates, with 32 transducers at a frequency of 1.1 MHz and water cooling. (**c**) The layer of the spheroid formation process for dedicated cancer cell lines. The prostate cancer cell line PC-3 and glioblastoma U87 were cultured in a dedicated medium using a liquid overlay technique to generate tumour spheroids.

Ultrasound is a safe, inexpensive, and widespread diagnostic tool capable of producing real-time, non-invasive images without evoking significant biological effects. In addition to ultrasound imaging, ultrasound waves can be focused at higher energies and sound pressures as therapeutic tools. Ultrasound can be generated by piezoelectric crystals, driven to vibrate by a specific fluctuating voltage. The devices containing these piezoelectric crystals and some electronics are called transducers since they convert electrical to mechanical energy and vice versa. In the case of focused ultrasound, the transducer creates ultrasound beams focused to a single focal zone, whereby the acoustic energy increases near and in the focus (Figure 1a).

FUS is a platform technology that produces biological responses through thermal or mechanical effects that act therapeutically on the target. These effects depend on the tissue composition (e.g., muscle vs. bone) and the ultrasound parameters (power, duration, mode—continuous vs. pulsed). The most pronounced effects caused by FUS on tissue are thermal ablation and mechanical tissue destruction (cavitation). The first one is a consequence of heating the tissue that denatures proteins and leads to the death of all cells, regardless of whether they are normal or abnormal. The dose required to produce irreversible damage and coagulative necrosis depends on the cell type, temperature, and duration of exposure—from 1 s at 56 °C to 240 min at 43 °C. Mechanical tissue destruction by FUS appears when the disruption of cells occurs through purely mechanical effects (no heating). The effect, called cavitation, occurs when gas bubbles oscillate in an ultrasonic field [18]. When these structures collapse, it is known as inertial cavitation, where enough force is accumulated to allow for targeted, localised tissue destruction.

Many other effects can be induced by FUS, such as sonoporation, vaso-dilation/constriction, substance delivery vehicles, or increasing vascular permeability, the clinical potentials of which are currently being investigated in, i.e., drug delivery, neuro-/immune-modulation, or radiation sensitisation.

For that reason, translation of FUS experiments performed on 3D tumour spheroids is necessary. It may also serve as a prudent act in avoiding animal experiments (replacement), limiting the number of animals (reduction) and their suffering (refinement) in tests to an absolute minimum [19]. The 3D tumour model (spheroid) development is essential to achieve the system’s benefits of mimicking avascular solid tumours. It provides more physiologically information when compared with 2D systems [20].

## 2. Materials and Methods

This study used a newly developed high-throughput in vitro FUS system to treat 3D spheroids in vitro.

### 2.1. Tumor Cell Lines and Cell Culture

The human prostate cancer cell line PC-3 was purchased from the ECACC (Salisbury, UK) and grown in Roswell Park Memorial Institute 1640 Medium (RPMI, gibco^®^ by Thermo Fisher Scientific (Waltham, MA, USA)). Human glioblastoma cell line U87 was obtained from the Department of Radiation Oncology (University of Leipzig, Leipzig, Germany). It was cultured in Dulbecco’s Modified Eagle’s Medium (DMEM, gibco^®^ by Thermo Fisher Scientific (Waltham, MA, USA)). All cell culture mediums were supplemented with 10% (*v*/*v*) fetal bovine serum (FBS, Sigma-Aldrich (St. Louis, MO, USA)) and 100 U/mL penicillin, and 100 mg/mL streptomycin (Biochrom GmbH (Berlin, Germany)) for culturing of the cell lines at 37 °C in a humidified incubator supplemented with 5% (*v*/*v*) CO_2_. The cells were routinely washed with phosphate-buffered saline without Ca^+^, Mg^+^, and phenol red (PBS BioWhittaker^®^, Lonza Group Ltd., (Basel, Switzerland)) and detached using trypsin/EDTA (Lonza Group Ltd., (Basel, Switzerland)).

### 2.2. Generation of PC-3 and U87 Spheroids

Following cell detachment, the dissociation enzyme was neutralised with a dedicated medium and the cells were centrifuged at 300× *g* for 5 min. The supernatant was removed, and the cell pellet was resuspended again in a complete growth medium. Cells were then counted with a Neubauer counting chamber (Paul Marienfeld GmbH & Co. KG (Lauda-Königshofen, Germany)) to achieve 5000 cells/200 μL for PC-3 and 1000 cells/200 μL for U87, respectively. A liquid overlay spheroid formation technique was chosen, adapted by Froehlich et al. [21], and a dedicated cancer cell line suspension of 200 μL was added to each well of ultra-low attachment (ULA) 96-well CELLSTAR^®^ round-bottom plates (Greiner Bio-One GmbH (Frickenhausen, Germany)). The cells were cultivated for four days in a humidified incubator (Figure 1c).

### 2.3. Characterisation of the In Vitro Applicator

The output was characterised before using the cell applicator for FUS treatment experiments on cell cultures. For this purpose, three sets of characterisation experiments were performed. First, the sound field of an individual transducer element was assessed to ensure that the sound fields from adjacent transducers did not overlap. Second, the output of all transducers was evaluated to characterise the homogeneity of the applicator. Finally, the acoustic output as a function of the applied power settings was measured for a single transducer element.

For assessment of the transducer performance, sound field measurements were performed in a water tank. The generated acoustic signals were acquired by a calibrated hydrophone (Type S, RP Acoustics (Leutenbach, Germany)) and analysed offline using Matlab (The MathWorks (Natick, MA, USA)). The hydrophone was moved in 2D to acquire the pressure distribution fields using an in-house-developed sound field scanning system.

An XY scan was performed in front of the whole applicator using the hydrophone in a second step. For data analysis, the surface corresponding to one well cross-section was automatically segmented in front of each transducer, and the intensity (I_SPTA_) was averaged within the segmented area.

### 2.4. Establishment of FUS Treatment of Tumour Cell Spheroids

The FUS in vitro system (Figure 1b) consists of a newly developed customised ultrasound cell applicator (Fraunhofer IBMT, (St. Ingbert, Germany)) designed for delivering acoustic energy to a 96-well cell culture plate. The applicator includes 32 cylindrically focusing transducers working at a frequency of 1.1 MHz. The 32 individual transducers are driven by a high power generator/amplifier (AG 1016, T&C Power Conversion Systems (Rochester, NY, USA)). The transducer can either be driven in parallel or in two subgroups of 16 transducers (each consisting of two rows of 8 transducers for sonication of one row of the well plate). An impedance matching circuit (T1K-7A, Power Conversion Systems) is connected between the applicator and the generator for improved efficiency. The transducers are water-cooled to prevent heat damage with an external pump (WK 16-1 DS, Colora Messtechnik GmbH, (Lorch, Germany)). An acoustically transparent membrane seals the cooling circuit. A water stand-off achieves acoustic coupling between the membrane and the well plate. A 3D-printed well-plate adapter was used to hold the well plate in the correct lateral and axial position. For control of the cell applicator, the customised software tool “Cell Therapy Planning Tool” (Fraunhofer IBMT (St. Ingbert, Germany)) was used. The software allows programming of the sonication time, the duty cycle, the burst repetition rate, and the applied power level, either for the whole applicator or for one of the two subgroups of 16 transducers.

It was essential for the experiment to prevent cells and the system from excessive heating and achieve merely mechanical acoustic effects. The real-time temperature in the wells during FUS treatment was monitored using an infrared thermal camera PI450 (Optris GmbH (Berlin, Germany)) and imaging software (PI Connect v2.16, https://www.optris.global/optris-pix-connect (accessed on 15 March 2020)).

### 2.5. Experimental Protocol for In Vitro Focused Ultrasound Treatment

Spheroids for each cancer cell line were formed as previously described. The spheroid selection was conducted to choose the most appropriate ones for the experiment (exclusion: externally-sourced cotton fibres and any other disintegrating factor) or for substitution in case of mishandling (pre-treatment stage only). For the execution of in vitro FUS treatment, the spheroids for each cancer cell were recruited. The remaining ones were devoted to negative (untreated group) and positive control (+5% DMSO) groups creation. The 96-well ultrasound penetrable μclear plate wells were filled with 150 μL of the dedicated cancer cell line medium and prepared + control solution medium (dedicated culture medium +5% DMSO). Then, 150 μL medium was removed from the spheroid-containing U-bottom plates, and the content (50 μL medium + spheroids) was transferred to the 96-well μclear plates. Before sonication, the 96-well μclear plates were sealed with paraffin titer top films (Electron Microscopy Sciences (Hatfield, PA, USA)) to prevent contamination of the wells and air bubble formation during FUS treatment. Water was placed on top of the foil above the transducers for coupling. The sealed well plate was slid into the yellow drawer until the aligned position with the transducer was reached, and where knobs ensured its ideal position. A visual inspection was performed to ensure that there is no air bubble between the well plate bottom and the membrane. The experiment proceeded with software settings for the desired parameters: 1st treatment parameter—sonication time: 90 s, power: 20% (I_SPTA_=2.95 W/cm^2^), signal repetition: 5 Hz (20 ms), duty cycle: 10%; 2nd treatment parameter—sonication time: 90 s, power: 40% (max) (I_SPTA_=~5.9 W/cm^2^), signal repetition: 5 Hz (20 ms), duty cycle: 10%. After sonication, the entire content of each well of 96-well μclear plate was transferred to corresponding wells of the U-bottom plate volume for further spheroid cultivation and follow-up. Cell culture medium with 5% DMSO of the positive control group was removed and refilled with ~200 μL/well fresh medium. The spheroids were then incubated for a further 48 and 96 h.

### 2.6. Microscopical Analysis of Spheroids

Morphological characteristics of formed spheroids were evaluated and recorded using microscopy (ZEISS Axio Observer, Carl Zeiss microscopy GmbH (Jena, Germany)) before and immediately after FUS treatment, and at 48 and 96 h after treatment. Pictures were taken with an AxioCam camera using ZEN v3.1 (blue edition) https://www.zeiss.com/microscopy/int/products/microscope-software/zen.html (accessed on 18 March 2020). Microscopy-acquired images of the spheroids were further processed and analysed with Java-based open-source software project ImageJ v1.53, https://imagej.nih.gov/ij/ (accessed on 10 May 2020), and MATLAB-based (© The MathWorks, Inc. (Natick, MA, USA)) open-source software AnaSP v1.4, https://sourceforge.net/projects/anasp/ (accessed on 8 May 2020) and ReViSP v2.2, https://sourceforge.net/projects/revisp/ (accessed on 8 May 2020).

### 2.7. Measurement of Cellular Metabolic Activity

A viability assay was performed at 48 and 96 h post-treatment to determine the metabolic activity of the cell spheroids. According to the manufacturers’ instructions, 150 μL of the medium was removed from each well of the 96-well clear F-bottom black plate (Greiner Bio-One GmbH (Frickenhausen, Germany)) and 50 μL of CellTiter-Glo^®^ 3D Reagent (Promega GmbH (Madison, WI, USA)) was added. Spheroids were then incubated at room temperature for 30 min in the dark to stabilise the bioluminescent signal, which was then measured using the Synergy H1™ Hybrid Multi-mode plate reader (BioTek Instruments^®^, Inc. (Winooski, VE, USA)).

### 2.8. Determination of DNA Double-Strand Breaks (DSBs) in PC-3 Spheroids

DSBs determination was performed by the γH2A.X assay 1 and 24 h post-treatment. Cell culture medium was aspirated from each well, and PC-3 cancer cell line spheroids were washed twice with PBS prior to disaggregation into single-cell suspension with trypsin (Lonza Group Ltd. (Basel, Switzerland)). Disaggregated spheroids were pooled in falcon tubes (TPP Techno Plastic Products AG (Trasadingen, Switzerland)), neutralising the enzyme by adding a dedicated cell culture medium and centrifuging. After medium aspiration, cells were fixed with 4% formaldehyde at 37 °C for 10 min and chilled on ice for 1 min. Afterwards, the fixative was removed, and cells were washed twice with PBS. Cells were permeabilised with 90% methanol on ice for 30 min, and again washed twice with a non-specific antibody binding blocking agent—0.5% bovine serum albumin (BSA, Cell Signalling Technology (Danvers, USA), 100 μL/well) in PBS. After removing the block solution (0.5% BSA in PBS), cells were incubated with phospho-histone H2A.X (Ser 139) rabbit primary monoclonal antibody (Cell Signalling Technology (Danvers, MA, USA)) at a concentration of 1:500 diluted with block solution at room temperature for 1 h. Cells were washed twice with 0.5% BSA in PBS solution and incubated with secondary antibody (anti-rabbit IgG conjugated with Alexa Fluor^®^ 488 fluorescent dye; Cell Signalling Technology (Danvers, MA, USA)) at a concentration of 1:1000 diluted with block solution at room temperature for 30 min in the dark. Cells were then washed twice with block solution, and cells were incubated with RNAse A (Sigma-Aldrich (St. Louis, MO, USA)) at 37 °C for 20 min. Finally, propidium iodide (Invitrogen by Thermo Fisher Scientific (Waltham, MA, USA)) was added and DSBs measurements were performed using Attune™ NxT flow cytometer (Thermo Fisher Scientific (Waltham, MA, USA)).

### 2.9. Statistical Analysis

Statistical analysis was performed using the statistical program Origin (Origin v6.0, https://www.originlab.com/origin (accessed on 21 June 2020) (Northampton, MA, USA)). All data of spheroid morphology analysis, cellular metabolic activity, spheroid cell hypoxia, and DNA double-strand breaks (γH2A.X) are expressed as means ± standard deviation of three independent experiments with three replicates, respectively. A one-way ANOVA test assessed the significance of the difference between the two mean values. A *p*-value ≤ 0.05 was considered to be statistically significant.

## 3. Results

### 3.1. Characteristics of the FUS In Vitro System

**Figure 2:**
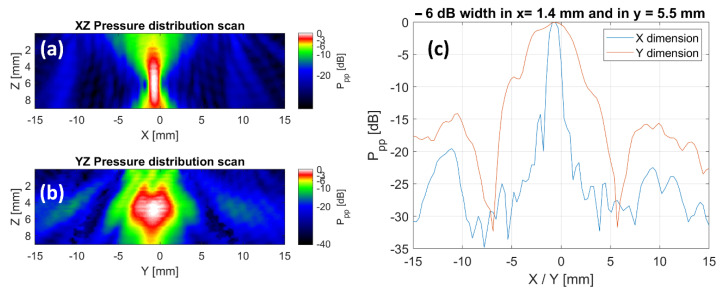
Assessment of the single-transducer pressure distribution in 2D and 1D. (**a**,**b**) show two 2D sound fields (Peak-to-Peak pressure in dB is plotted) acquired in a water tank measurement to assess the extent of the focal area. (**c**) Shows a lateral cross-section through the depth of highest pressure.

The obtained 2D and 1D pressure distribution fields are shown in Figure 2, in which the Peak to Peak pressure (scaled in dB) is plotted. The −6 dB focal widths in the x- and y-directions are 1.4 mm and 5.5 mm, respectively. This is below the pitch of the well plate (in both dimensions), confirming that there is little influence on individual wells by neighbouring transducers.

**Figure 3:**
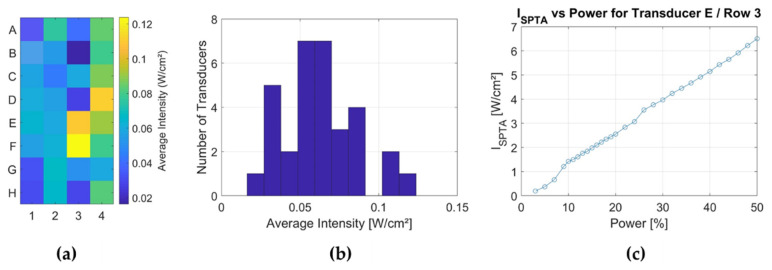
Assessment of the applicator performance. An XY sound field scan was performed in front of the cell applicator. For (**a**), the acoustic intensity I_SPTA_ was averaged over the surface of one well of the 96-well plate in front of each transducer element. (**b**) A histogram allowing assessing the homogeneity of the intensity output. (**a**,**b**) were performed with a power setting of 1% and were made to compare the relative performance of the different wells, not the absolute intensities. (**c**) The I_SPTA_ as a function of the power setting that can be user-defined using the “Cell Therapy Planning Tool”.

The corresponding average intensity for each well is shown in Figure 3a. In this experiment, the generator was driven with a power of only 1% so that the measurement can only be taken for the relative comparison of the intensity output from well to well and not for the absolute maximum intensity. A histogram of the average I_SPTA_ is given in Figure 3b. Finally, for one transducer element (E3), the I_SPTA_ in the focus was measured as a function of the power level defined in the “Cell Therapy Planning Tool”. In this analysis, a duty cycle (DC) of 100% was assumed such that the I_SPTA_ equals the I_SPPA_.

### 3.2. Mitigation of Spheroid Growth

**Figure 4:**
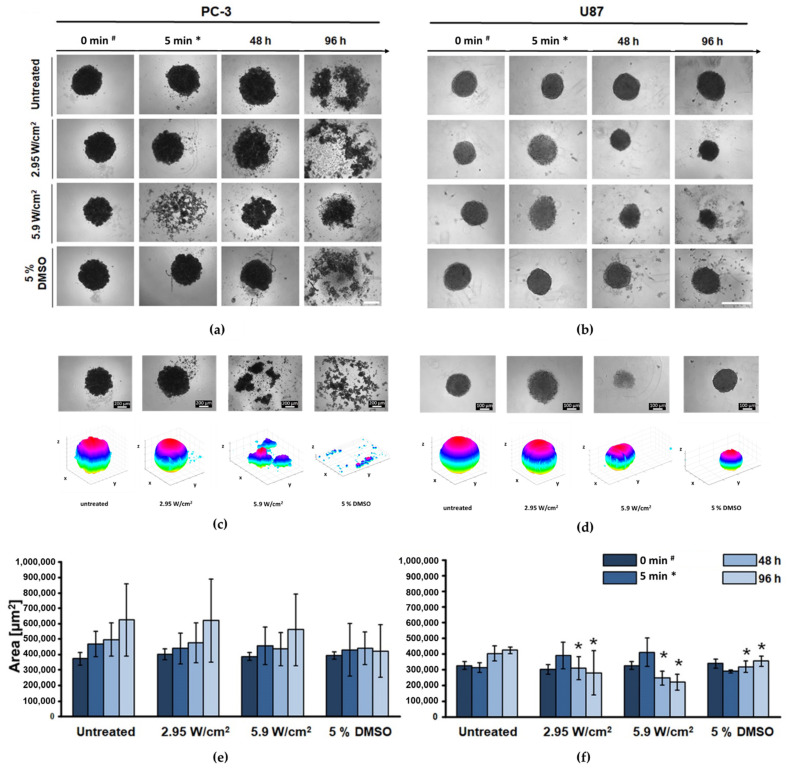
FUS reduced spheroid size and led to a loss of integrity. (**a**,**b**) Representative microscopy images showing alterations in spheroid morphology and (**c**,**d**) brightfield images of FUS-treated spheroids; the corresponding 3D reconstructions were obtained using ReViSP, http://sourceforge.net/p/revisp/ (accessed on 8 May 2020). (**e**/**f**) Bar chart representation of changes in the spheroid area before, immediately, 48, and 96 h after FUS treatment at an intensity of 2.95 and 5.9 W/cm^2^, + control: +5% DMSO. Data analysis was carried out by one-way ANOVA. * Significantly different from the untreated group. (*p* ≤ 0.05). # 4th day spheroid formation; * in the ‘untreated’ group only, well-to-well (re-)transfer of spheroid was carried out to equalise the impact. PC-3 cancer cell line: Scale bar = 200 μm. U87 cancer cell line: Scale bar = 100 μm. *n* = 9.

Microscopy analysis of the spheroid morphology showed decomposition of the PC-3 spheroids immediately after FUS treatment at 5.9 W/cm^2^. Interestingly, the spheroid reassembles 48 h post-treatment. Depending on time and thus an absence of nourishment, the PC-3 spheroids lost their integrity at 96 h in the untreated control group (Figure 4a). In contrast, the U87 spheroids had a more tightened spheroid structure, and growth of the spheroid structure was detected in the untreated group. Morphologically, there was no loss of spheroid structure apparent in the U87 cell line in all treatment groups. However, significant changes (*p* ≤ 0.05) of the measured spheroid area in U87 cancer cell line spheroid were noticed 48 and 96 h after treatment, with a reduction of the area to 246,387 µm^2^ and 219,976 µm^2^ after 5.9 W/cm^2^, respectively, as compared with the negative control. A slight swelling of the spheroid size immediately after sonication was determined in U87 spheroids (Figure 4b).

### 3.3. Diminished Spheroid Cell Viability

**Figure 5:**
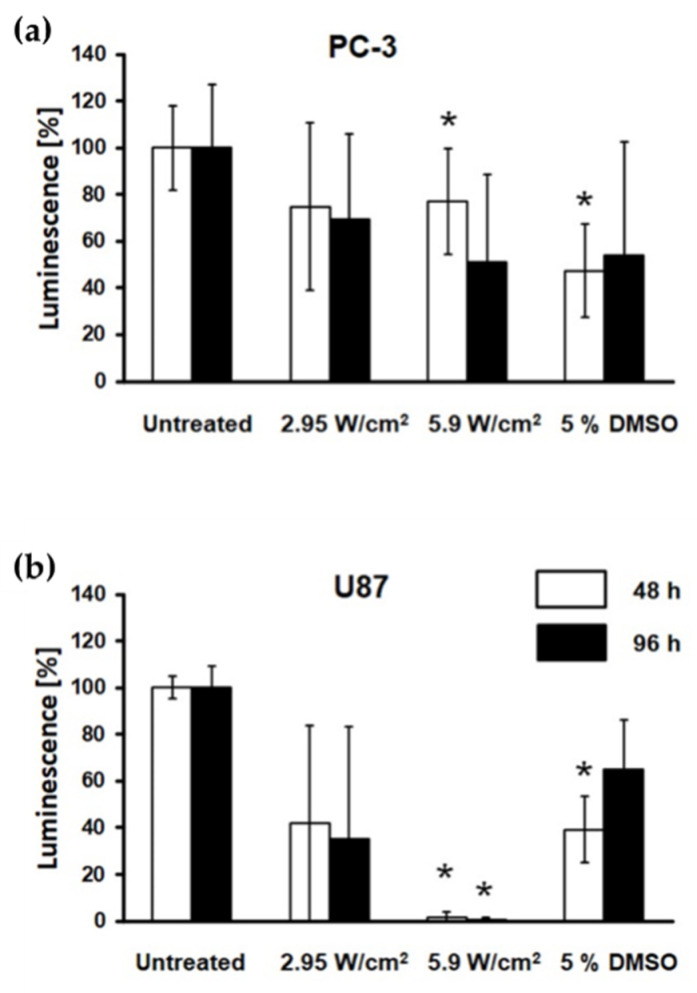
FUS diminished spheroid metabolic activity. ATP content of PC-3 (**a**) and U87 (**b**) spheroids was assessed using CellTiter-Glo^®^ 3D Cell Viability assay 48 and 96 h after treatment, showing the reduction of cell metabolic activity. Data sets were normalised to the untreated control group (100%), while data analysis was carried out by one-way ANOVA. * Significantly different from the untreated group (*p* ≤ 0.05). *n* = 9.

To evaluate the effects of FUS treatment on spheroids, cellular metabolic activity was checked 48 and 96 h post-treatment. The FUS treatment reduced the cell metabolic activity in both cell lines in an intensity-dependent manner (Figure 5). This reduction below 80% of viability was pronounced 48 and 96 h after the treatment compared with the untreated control (Figure 5). The glioblastoma cell line U87 showed a higher sensitivity with a statistically significant loss in ATP metabolism after treatment at 5.9 W/cm^2^ (Figure 5b) to 1.61 ± 2.45% (48 h) and 0.70 ± 0.94% (96 h) in U87 cells (Figure 5b).

### 3.4. FUS Enhanced Spheroid Cell DNA Damage

**Figure 6:**
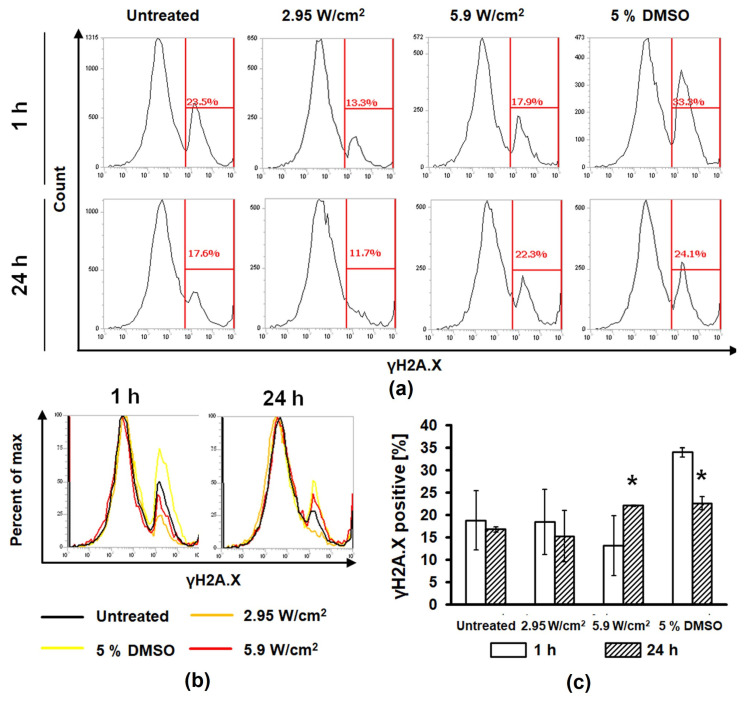
FUS treatment enhanced the number of DNA double-strand breaks after 24 h. (**a**) Representation of *γ*H2A.X percentages as a function of cell count determined by gating histograms derived from dissociated PC-3 spheroids with flow cytometric analysis 1 and 24 h post-treatment. (**b**) Overlayed flow cytometry images (**c**) and quantified results show an increasing number of *γ*H2A.X positive cells 24 h after 5.9 W/cm^2^. Data analysis was carried out by one-way ANOVA. * Significantly different from negative control (*p* ≤ 0.05).

The γH2A.X assay was performed 1 and 24 h post-treatment to explore the potential of FUS to affect DNA repair. Flow cytometer analysis of dissociated PC-3 spheroids revealed a significant increase of fluorescence intensity (*p* ≤ 0.05) 24 h post-treatment with FUS at an intensity of ~5.9 W/cm^2^ and 5% DMSO groups, 22.1 ± 0.18% and 22.62 ± 1.49%, respectively (Figure 6). No significant increase of DNA double-strand breaks after FUS treatment was observed 1 h after treatment.

## 4. Discussion

While most of the studies evaluating the effect of FUS treatment in vitro utilise the 2D model, with the cancer cells distributed as a monolayer, our research aimed to test a new developed FUS in vitro system for high-throughput sonication as a starting point to establish the effect of FUS on the tumour entity, using 3D tumour culture. The difference in sphere-forming potential of the selected cancer cell lines was revealed with the predisposition of GBM (U87) spheroid to be regular in shape constituting tight cell-cell adhesions, while prostate cancer (PC-3) resembled more of a roundly-arranged, still irregular, tumour cell aggregate. This peculiar characteristic might be the reason behind the noticeable, but statistically insignificant, reduction of the PC-3 spheroid size after FUS treatment in all experiments. However, it significantly manifested in the case of U87 spheroid, suggesting that the response of the specific cancer cell’s type cluster to the acoustic constrain might be determined by its biophysical properties. This phenomenon was described in previous studies [22], indicating heightened resistance of spheroids over the standard flask cultures to radiation and chemotherapeutics, an aspect articulating the necessity to modify the research design with various cancer types in analogous future experiments.

Since our newly developed FUS in vitro system (2nd gen. cell applicator) was applied and no existing studies for FUS on spheroids were available, the system’s setup and FUS parameters needed to be established first. For acoustic characterisation of the FUS applicator, various measurements were performed by our partner, Fraunhofer IBMT, the manufacturer of the system. The results stated that the intensity distribution was relatively homogeneous within distinct well outliners, while I_SPTA_ varied with the increase of the power setting to its maximum (40%) starting from the power at the level of 12%, with the need of its (I_SPTA_) extrapolation. It resulted in the approximation of the 2nd treatment parameter to ~5.9 W/cm^2^, which may not reflect the identical acoustic wave intensity distribution across all wells, which is the greatest limitation of this FUS setup. Adaption of the FUS intensity parameter sets (~3–6 W/cm^2^) was cerebrated depending on a recently conducted study [23], revealing that using a low-intensity pulsed ultrasound (LIPUS) with acoustic intensity similar to that of diagnostic levels, pulse duration of greater than 10 ms induces cyto-disruption. Intriguingly, what was noticed in this study was the expansion in the size of U87 spheroids right after the FUS treatment. It may be questioned whether the acoustic exposure on the spheroids evoked dismantling of its cell-cell architecture leading to its cyto-disruptive collapse and whether the same phenomenon may occur in more solid conditions, where tumour cells are embedded within surrounding tissue. Indeed, future in vivo studies are needed to test this hypothesis.

CellTiter-Glo^®^ 3D Cell Viability Assay [17] using ATP-dependent luminescence signal quantification to determine the number of viable cells in 3D cell culture was the best method in this study to determine the metabolic activity in physical treatments. This ATP detection assay is currently the most sensitive and has minimal interferences [24]. While FUS treatment induced a significant reduction of viable cells in both tumour spheroids over time with plate reader estimation, specialised techniques of spatial-temporal quantification [25] might bring more relevant data. The significant reduction of the spheroid area in U87 spheroids is congruent with the significant loss in cellular ATP metabolism showing the collapse of U87 cells. Furthermore, the short loss of the spheroid structure of PC-3 immediately after FUS seems to have no impact on the ATP metabolism, only on the binding, e.g., tight junctions, between the cells. The detected decrease of ATP content in PC-3 was not reflected in the observed reduction of the spheroid area within the 96 h time frame.

Measurement of the DNA double-strand breaks, which can be used as a marker for treatment-induced cell death in tumours [16], showed an increased fluorescence intensity of immunolabelled γH2A.X in cells of PC-3 spheroids after FUS treatment with the intensity of ~5.9 W/cm^2^, revealing a damaging effect of pulsed FUS, like the cytotoxic agent 5% DMSO used in the positive control group. Our study proved, in 24 h post-treatment, that the FUS treatment accelerated notably the DNA damage in PC-3 spheroids, leading to cell death. On the other hand, U87 spheroids could not be completely dissociated with the methods (trypsinisation) used in the case of PC-3 spheroids.

## 5. Conclusions

The present work highlighted the potential of FUS application for the treatment of tumour spheroids. It was demonstrated that low-intensity pulsed focused ultrasound reduced spheroid growth metabolic activity and increased DNA double-strand breaks in particular cancer cell lines used in this study. The results suggest that FUS treatment in LIPUS mode harm cancer cells and the modality itself has great potential to be further investigated in vivo.

## Data Availability

Not applicable.
